# Supplementary material for: Investigating Potential Cardiovascular Toxicity of Two Anti-Leukemia Drugs of Asciminib and Ponatinib in Zebrafish Embryos
Source: Int J Mol Sci. 2022 Oct 3;23(19):11711. doi: 10.3390/ijms231911711 (PMC9570146; doi:10.3390/ijms231911711)
Supplement: Supplementary file 1 [file ijms-23-11711-s001.zip › ijms-1892300-supplementary-tables and figures.pdf]

Table S1. List of primer sequences used for qRT-PCR.

Gene name	Sequence of forward primer (5′ – 3′)	Sequence of reversed primer (5′ –3′)
myh6	F: CACCAGCAGACACTGGATG	R: GCTCCAAGTCCATTCTGAC
tbx5	F: ATTCGCCGATAACAAATGG	R: CGCCTTGACGATGTGGAT
vmhc	F: GAAGAGGCAGAGGCATCACT	R: AATTGCGTTTGCTCTGCTCC
nxk2.5	F: GTCCAGGCAACTCGAACTACTC	R: AACATCCCAGCCAAACCATA
amhc	F: AAGCCACTACCGCCTCTCTA	R: TTTGAGGCAAGGTCGTCCAA
vegfaa	F: AAAAGAGTGCGTGCAAGACC	R: GACGTTTCGTGTCTCTGTCG
vegfab	F: TGTTGGTGGAAATTCAGCAG	R: CACCCTGATGACGAAGAGGT
hbbe1	F: GCTCTGGCAAGGTGTCTCAT	R: TTCTTCACTGCCAGCTCCAG
hbbe2	F: ACTATGAGGAGGCTGGAC	R: CGGCGTAGGTGTTCTTG
hbae1	F: CCAGGATGTTGATTGTCTAC	R: CAGTCTTGCCGTGTTTC
gata1	F: GAGACTGACCTACTGCCATCG	R: TCCCAGAATTGACTGAGATGAG
fli1	F: CAACGGATCCAGAGAGTCG	R: CCATGTAGCCAGTATAGTTCATCTG

Table S2. List of target proteins and grid box parameters for molecular docking studies.

Target Proteins/ PDB ID	Grid box parameters (x, y, z coordinates)
ABL1 (5MO4)	Center: x=-45.339, y= 23.060, z= 8.080<br>Dimensions (Å): x= 20.037, y= 17.067, z= 24.016
Mutated ABL1 (4TWP)	Center: x= 59.372, y= 14.989, z= 49.426<br>Dimensions (Å): x= 15.226, y= 17.945, z= 15.059
ABL2 (3HMI)	Center: x= -26.618, y= 9.508, z= -13.074<br>Dimensions (Å): x= 22.366, y= 18.956, z= 14.232
Mutated ABL2 (2KK1)	Center: x= -9.040, y= 4.417, z= -7.919<br>Dimensions (Å): x= 42.961, y= 51.842, z= 47.421

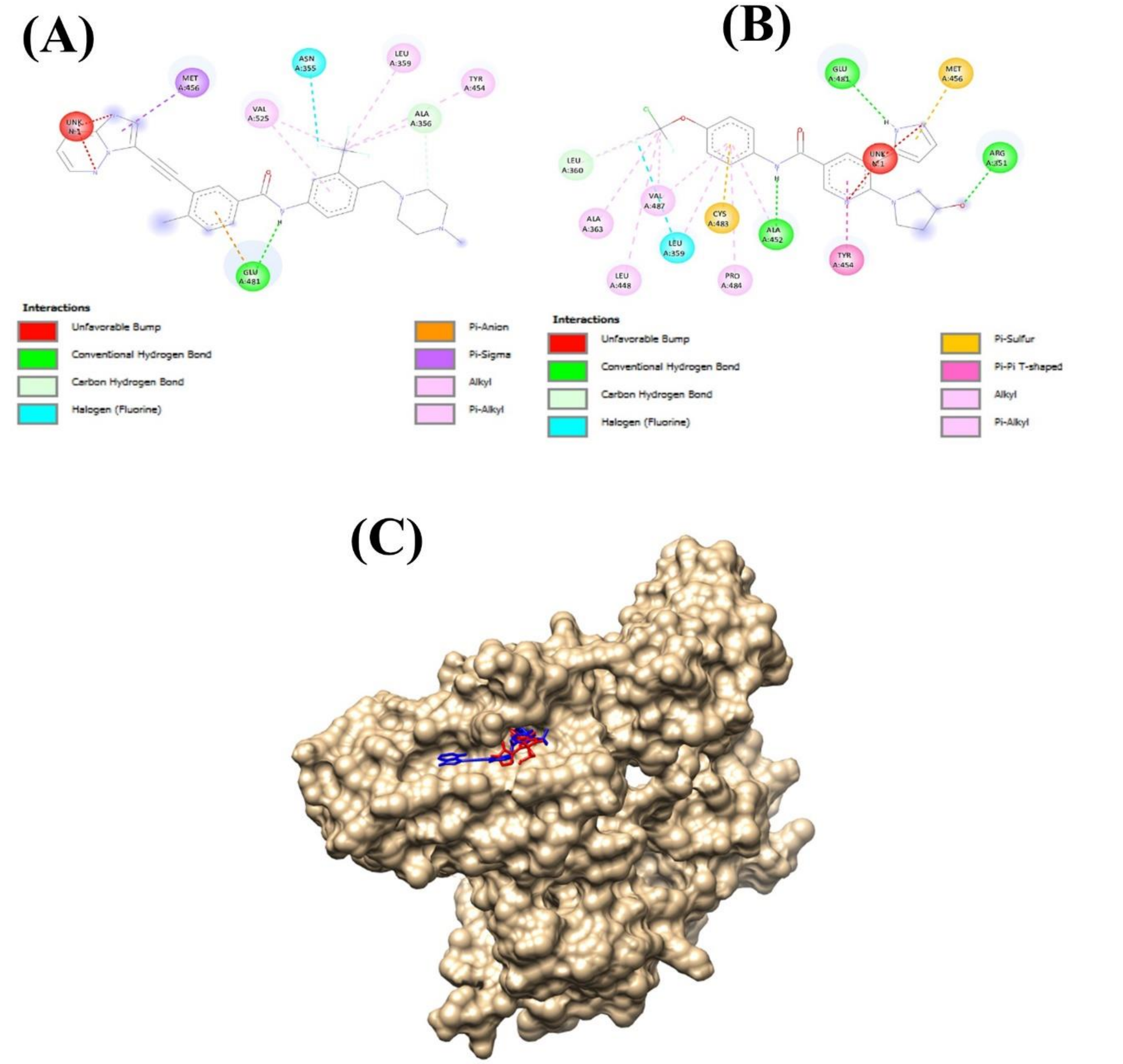

Figure S1. Molecular docking of Ponatinib and Asciminib to BCR-Abl1 normal protein (5MO4). **(A)** 2D interaction diagram of ponatinib with ABL1; (**B**) 2D interaction diagram of asciminib with ABL1; (**C**) 3D docking snapshot with ponatinib (blue) and asciminib (red) on the surface of protein.

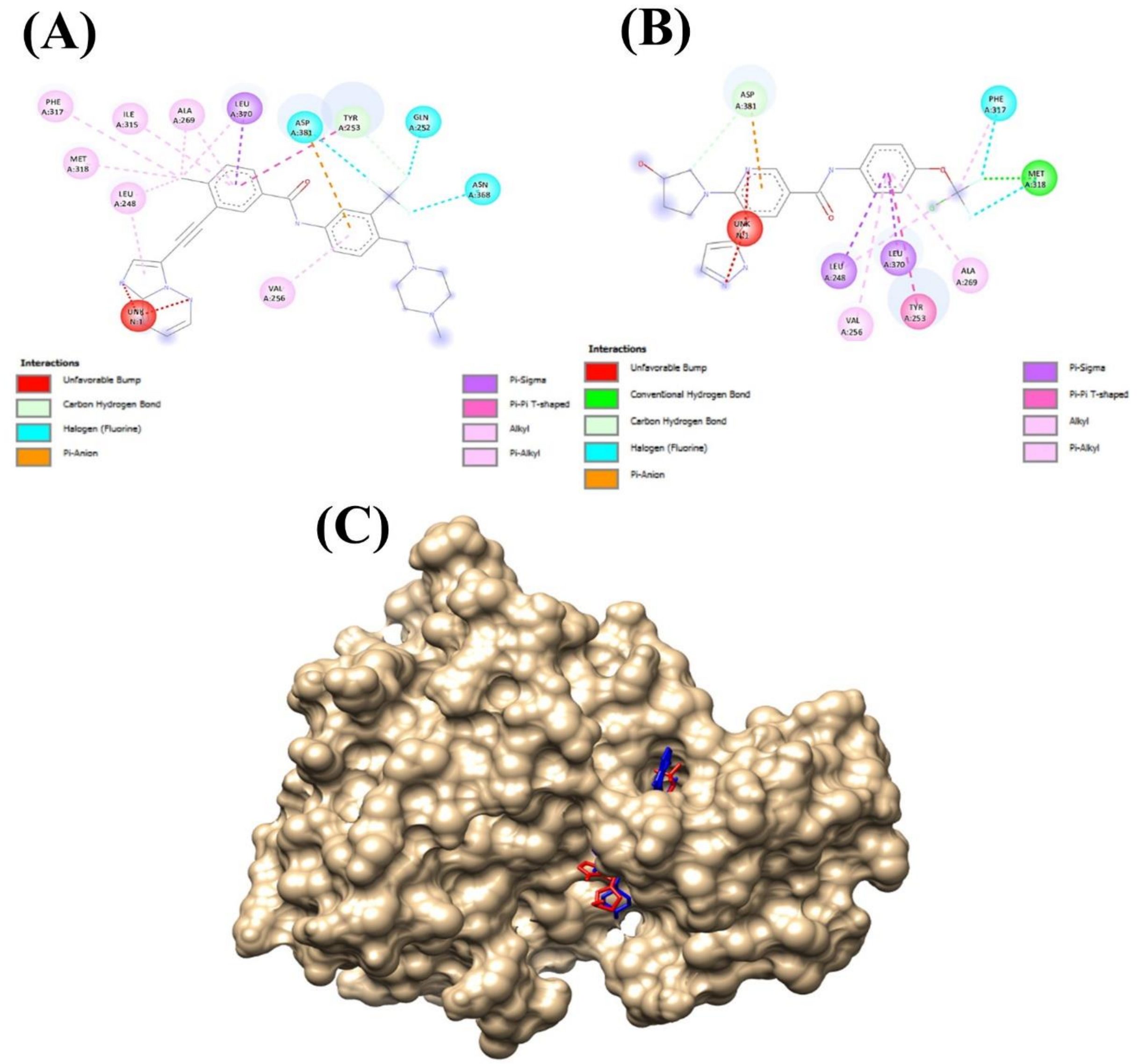

Figure S2. Molecular docking of Ponatinib and Asciminib to BCR-Abl1 mutated protein (4TWP). **(A)** 2D interaction diagram of ponatinib with ABL1; (**B**) 2D interaction diagram of asciminib with ABL1; (**C**) 3D docking snapshot with ponatinib (blue) and asciminib (red) on the surface of protein.

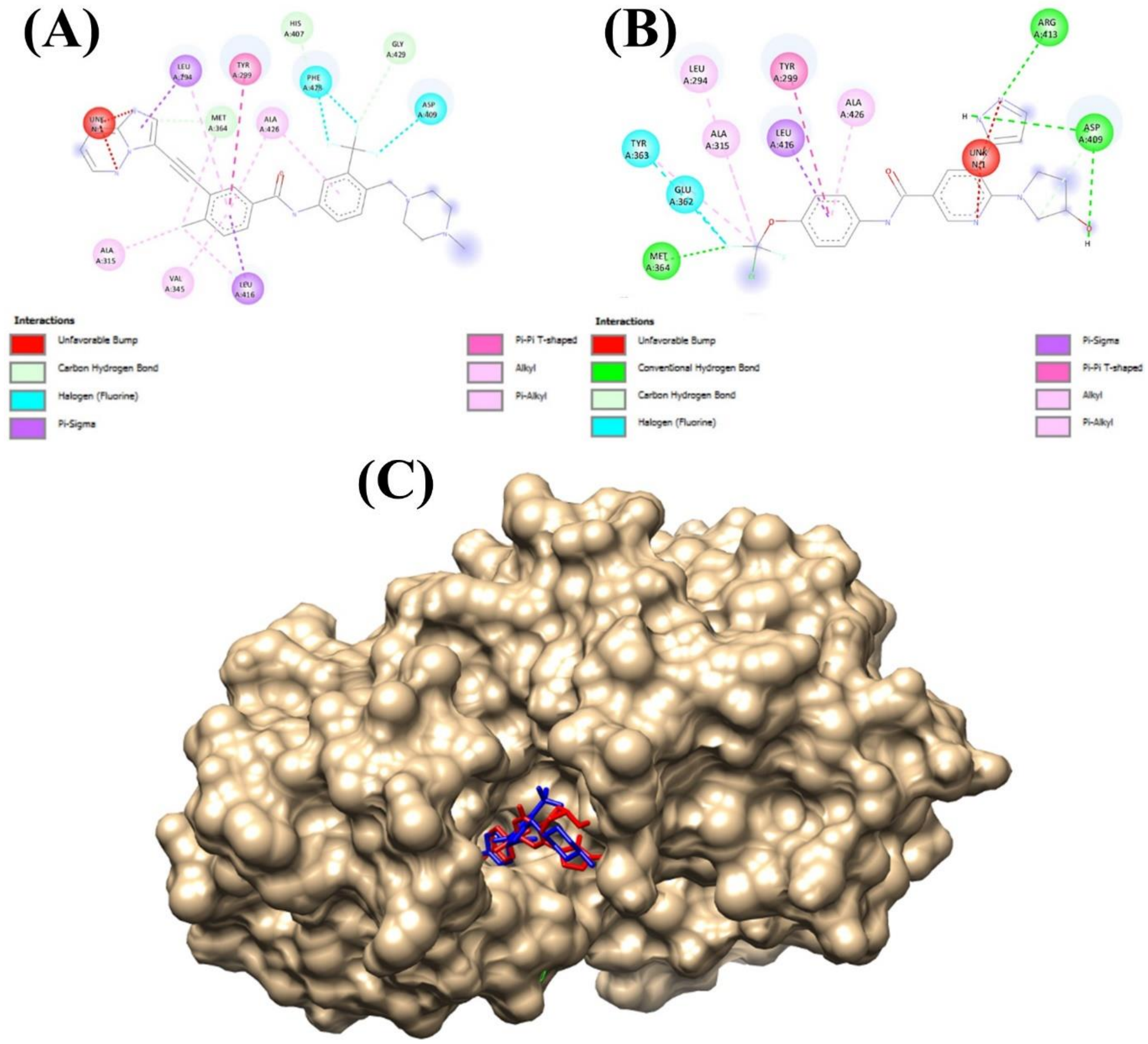

Figure S3. Molecular docking of Ponatinib and Asciminib to BCR-Abl2 normal protein (3HMI). **(A)** 2D interaction diagram of ponatinib with ABL1; (**B**) 2D interaction diagram of asciminib with ABL1; (**C**) 3D docking snapshot with ponatinib (blue) and asciminib (red) on the surface of protein.

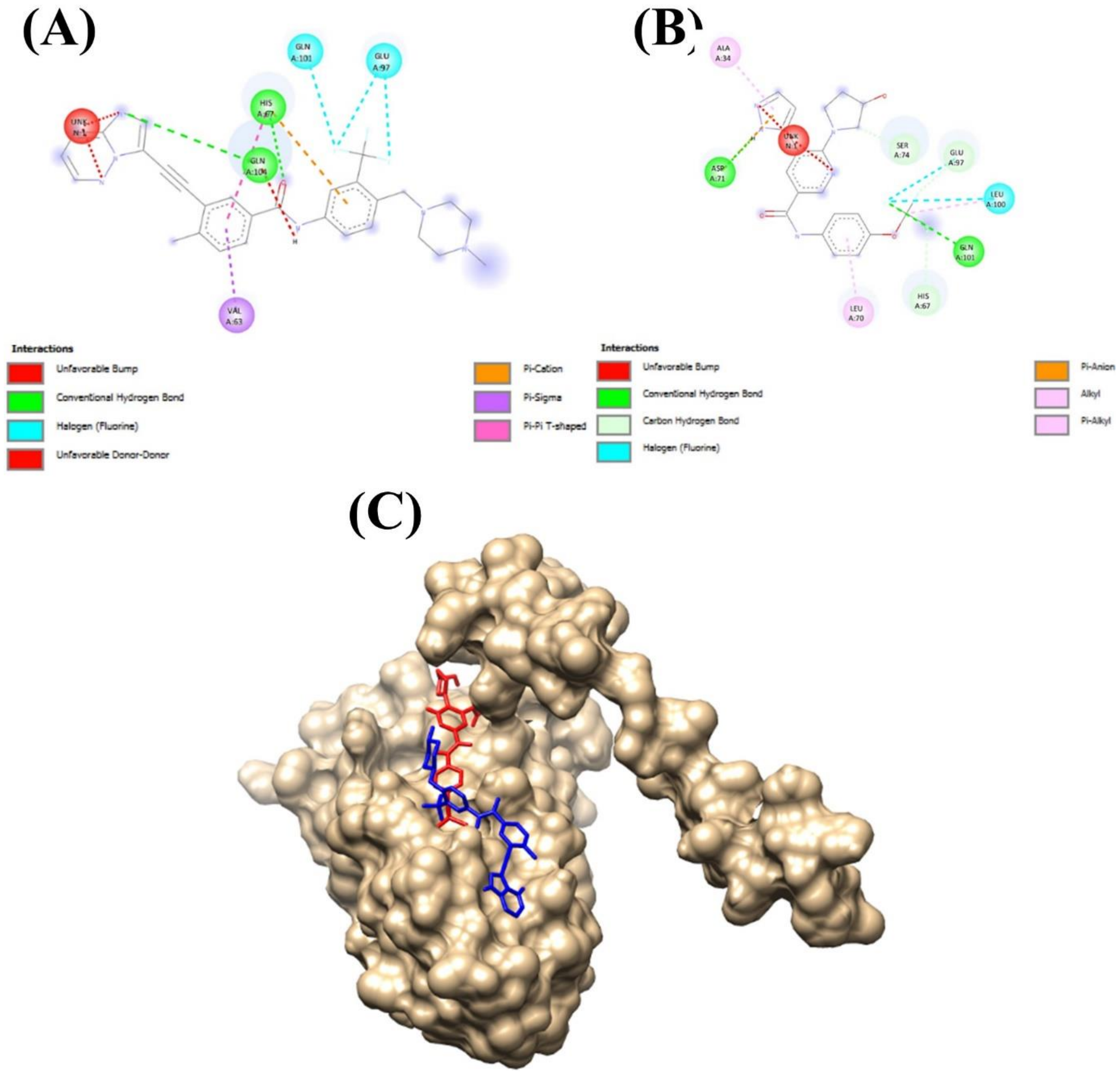

Figure S4. Molecular docking of Ponatinib and Asciminib to BCR-Abl2 mutated protein (2KKI). **(A)** 2D interaction diagram of ponatinib with ABL1; (**B**) 2D interaction diagram of asciminib with ABL1; (**C**) 3D docking snapshot with ponatinib (blue) and asciminib (red) on the surface of protein.
